# Sensor Fusion Based Model for Collision Free Mobile Robot Navigation

**DOI:** 10.3390/s16010024

**Published:** 2015-12-26

**Authors:** Marwah Almasri, Khaled Elleithy, Abrar Alajlan

**Affiliations:** Computer Science and Engineering Department, University of Bridgeport, 126 Park Ave, Bridgeport, CT 06604, USA

**Keywords:** autonomous mobile robots, collision avoidance, path following, fusion, fuzzy logic

## Abstract

Autonomous mobile robots have become a very popular and interesting topic in the last decade. Each of them are equipped with various types of sensors such as GPS, camera, infrared and ultrasonic sensors. These sensors are used to observe the surrounding environment. However, these sensors sometimes fail and have inaccurate readings. Therefore, the integration of sensor fusion will help to solve this dilemma and enhance the overall performance. This paper presents a collision free mobile robot navigation based on the fuzzy logic fusion model. Eight distance sensors and a range finder camera are used for the collision avoidance approach where three ground sensors are used for the line or path following approach. The fuzzy system is composed of nine inputs which are the eight distance sensors and the camera, two outputs which are the left and right velocities of the mobile robot’s wheels, and 24 fuzzy rules for the robot’s movement. Webots Pro simulator is used for modeling the environment and the robot. The proposed methodology, which includes the collision avoidance based on fuzzy logic fusion model and line following robot, has been implemented and tested through simulation and real time experiments. Various scenarios have been presented with static and dynamic obstacles using one robot and two robots while avoiding obstacles in different shapes and sizes.

## 1. Introduction

The area of autonomous mobile robots has gained an increasing interest in the last decade. Autonomous mobile robots are robots that can navigate freely without human involvement. Due to the increased demand of this type of robots, various techniques and algorithms are developed. Most of them are focused on navigating the robot in collision-free trajectories with the controlling of the robot’s speed and direction. The robot can be mounted by different kinds of sensors in order to observe the surrounding environment and thus steer the robot accordingly. However, many factors affect the reliability and efficiency of these sensors. The integration of multi-sensor fusion systems can overcome this problem by combining inputs coming from different types of sensors, hence have more reliable and complete outputs. This plays a key role in building a more efficient autonomous mobile robotic system. 

There are many sensor fusion techniques that have been proven to be effective and beneficial, especially in detecting and avoiding obstacles as well as path planning of the mobile robot. Fuzzy logic, neural network, neuro-fuzzy, and genetic algorithms are examples of well-known fusion techniques that help in moving the robot from the starting point to the target without colliding with any obstacles along its path.

Obstacles detected can be moving or static objects in known or unknown environments. In addition, the path planning behavior can be categorized as global path planning where the environment is entirely known in advance, or local path planning where the environment is partly known or not known at all. The latter case is called dynamic collision avoidance [1].

For the purpose of collision avoidance and path following approaches, different types of sensors such as camera, infrared sensor, ultrasonic sensor, and GPS can detect different aspects of the environment. Each sensor has its own capability and accuracy, whereas integrating multiple sensors enhances the overall performance and detection of obstacles. Many researchers have used sensor fusion to fuse data from various types of sensors, which improved the decision making process of routing the mobile robot. A hybrid mechanism was introduced by [2] which uses the neuro-fuzzy controller for collision avoidance and path planning behavior for mobile robots in an unknown environment. Moreover, an adaptive neuro-fuzzy inference system (ANFIS) was applied for an autonomous ground vehicle (AGV) to safely reach the target while avoiding obstacles by using four ANFIS controllers [3]. Another sensor fusion based on Unscented Kalman Filter (UKF) was used for mobile robots’ localization problems. Accelerometers, encoders, and gyroscopes were used to obtain data for the fusion algorithm. The proposed work was tested experimentally and was successfully capable of tracking the motion of the robot [4]. In [5], Teleoperated Autonomous Vehicle (TAV) was designed with collision avoidance and path following techniques to discover the environment. TAV includes GPS, infrared sensors, and the camera. Behavior based architecture is proposed which consist of obstacle avoidance module (OAM), Line Flowing Module (LFM), Line Entering Module (LEM), Line Leaving Module (LLM) and U-Turn Module (UTM). Sensor fusion based on Fuzzy logic was used for collision avoidance where neural network fusion was used for the line following approach.

This paper focuses on the integration of multisensory information from range finder camera and infrared sensors using Fuzzy logic fusion system for collision avoidance and line follower mobile robots. The proposed methodology develops membership functions for inputs and outputs and designs fuzzy rules based on these inputs and outputs. 

The rest of the paper is organized as follows: Section 2 presents the related work. Section 3 demonstrates the proposed methodology based on fuzzy logic system. Section 4 shows the simulation and real time implementations. Section 5 discusses the results in details. Section 6 concludes the paper.

## 2. Related Work

Many obstacle detection, obstacle avoidance, path planning techniques have been proposed in the field of autonomous robotic systems. This section presents some of these techniques with the collaboration of sensor fusion to obtain best results.

Chen and Richardson proposed a collision avoidance mechanism for mobile robot navigating in unknown environments based on a dynamic recurrent neuro-fuzzy system (DRNFS). In this technique, a short memory is used that is capable of memorizing the past and the current information for a more reliable behavior. The ordered derivative algorithm is implemented for updating the DRNFS parameters [6]. Another collision avoidance approach for mobile robots was proposed by [7], which is based on multi sensor fusion technology. With the use of ultrasonic sensors and infrared distance sensors, a 180° rolling window was established in front of the robot. The robot’s design has mostly focused on four main layers as follows: energy layer, driver layer, sensor layer, and, finally, the master layer [7].

In addition, a collision avoidance algorithm for a network-based autonomous robot was discussed in [8]. The algorithm is based on the Vector Field Histogram (VFH) algorithm with the consideration of the network’s delay. The system consists of sensors, actuators, and the VFH controller. Kalman filter fusion is applied for the robot’s localization in order to compensate for the delay between the odometry and environmental sensor readings [8]. 

The Kalman filtering fusion technique for multiple sensors has been applied in [9]. The Kalman filter is used for predicting the position and distance to the obstacle or wall using three infrared range finder sensors. Authors claimed that this technique is mostly helpful in robots’ localization, automatic robots’ parking, and collision avoidance [9].

Furthermore, in [10], a path control for mobile robots based on sensor fusion is presented where the deliberative/reactive hybrid architecture is used for handling the mobile robot motion and path control. The sensor fusion technology helps the robot to reach the target point successfully [10]. Another multi sensor fusion system was designed in [11]. This system was mainly for navigating coal mine rescue robots. It used various types of sensors such as infrared and ultrasonic sensors with digital signal processing. The multi-sensor data fusion system helped in decreasing errors caused by the blind zone of ultrasonic sensors [11].

Moreover, a transferable belief model (TBM) was applied in mobile robot for the purpose of a collision-free path planning navigation in a dynamic environment which contains both static and moving objects. TBM was used for building the fusion system. In addition, a new path planning mechanism has been proposed based on TBM. The main benefit of designing such mechanisms is the recognition of the obstacle’s type whether it is dynamic or static without the need of any previous information [12].

In [13], the authors developed a switching path-planning control scheme that helped in avoiding obstacles for a mobile robot while reaching its target. In this scheme, a motion tracking mode, obstacle avoidance mode, and self-rotation mode were designed without the need of any previous environmental information [13]. 

Another multi-sensor particle filter fusion based algorithm for mobile robot localization was proposed by [14]. The algorithm was able to fuse data coming from various types of sensors. Authors also proposed an easy and fast deployment mechanism of the proposed system. A laser range-finder, a WiFi system, many external cameras, and a magnetic compass along with a probabilistic and mapping strategy were used to validate the work proposed [14]. 

In [15], a novel multi-sensor data fusion methodology for autonomous mobile robots in unknown environments was designed. The flood fill algorithms and fuzzy algorithms were used for the robot’s path planning, whereas Principal Component Analysis (PCA) was used for object detection. Multiple sensor data were fused using Kalman Filter fusion technique from infrared sensor, ultrasonic sensor, camera, and accelerometer. The proposed technique has successfully reduced the time and energy consumption.

## 3. Proposed Methodology

This section presents the proposed methodology for mobile robot collision free navigation with the integration of the fuzzy logic fusion technique. The mobile robot is equipped with distance sensors, ground sensors, camera, and GPS. Distance sensors which are infrared sensors, and the camera are used for collision avoidance behavior where the ground sensors are used for path follower behavior. GPS is used to get the robot’s position. The goal of the proposed technique is as follows:

- The capability of the mobile robot to avoid obstacles along its path;
- The integration of sensor fusion using fuzzy logic rules based on sensor inputs and defined membership functions;
- The capability of the mobile robot to follow a predetermined path;
- The performance of the mobile robot when programmed with the fuzzy logic sets and rules.

### 3.1. Robot and Environment Modeling

Webots Pro simulator is used to model the robot and the environment. Webots Pro is a Graphical User Interface (GUI) which creates an environment that is suitable for mobile robot simulation. It also allows creating obstacles in different shapes and sizes. The mobile robot used in Webots Pro simulator is called E-puck robot which is equipped with a large choice of sensors and actuators such as camera, infrared sensors, GPS, and LED sensors [16]. 

The environment in this paper is modeled with a white floor that has a black line in order for the robot to follow it. It also has solid obstacles where the robot should avoid them. The environment in Webots Pro is called “world.” A world file can be built using a new project directory. Each project file composed of four main windows which are: the Scene tree which represents a hierarchical view of the world, the 3D window that demonstrates the 3D simulation, the Text editor that has the source code (Controller), and the Console that shows outputs and compilation [16].

**Figure 1:**
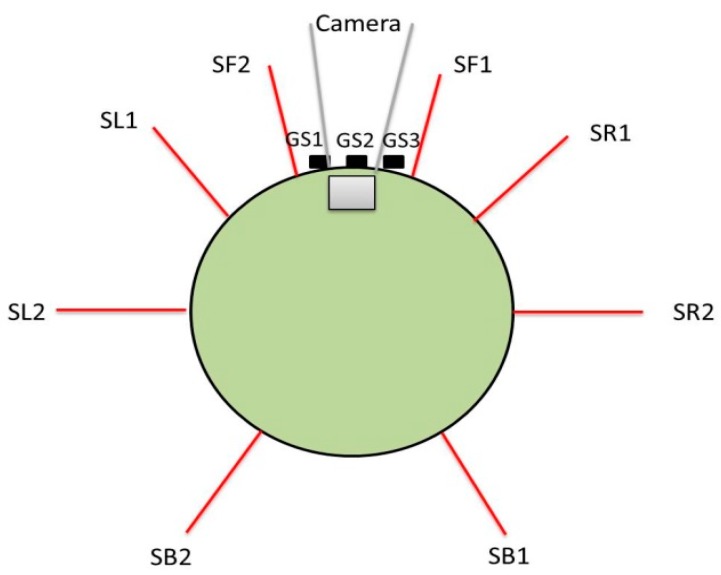
The top view of E-puck robot with various types of sensors.

The two differential wheel robot (E-puck robot) that is used in this paper is equipped with eight infrared sensors (distance sensors), a camera, and three ground sensors which are also infrared sensors. The eight distance sensors are used to detect obstacles. Each distance sensor has a range of 0 to 2000 where 0 is the initial value of the distance sensor, which means there is no obstacle detected. As the mobile robot approaches the obstacle, its value is increased accordingly. When an obstacle is detected, the distance sensor value will be 1000 or more depending on the distance between the sensor and the obstacle. The camera sensor that is used in this work is a range finder type of camera which allows obtaining distance in meters between the camera and the obstacle from the OpenGL context of the camera. Finally, the three ground sensors are located in front of the e-puck robot where all of them are pointing directly to the ground. These sensors are used to follow the black line drawn on the floor. Figure 1 shows the E-puck robot top view with different types of sensors.

### 3.2. Design of the Fusion Model

**Figure 2:**
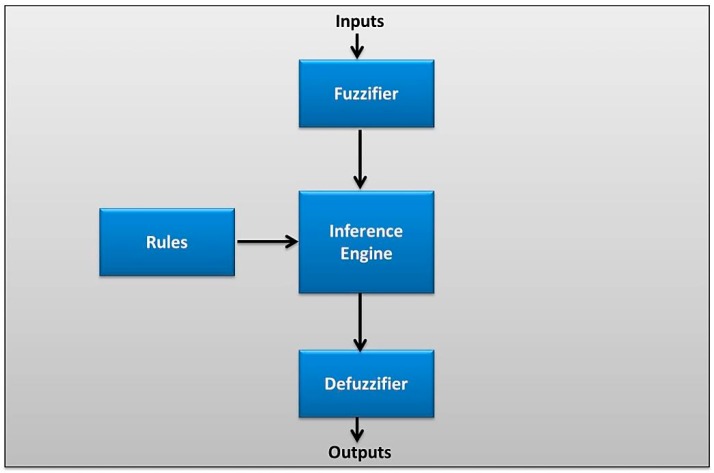
Block diagram of the Fuzzy Logic System.

Multisensory fusion model is designed for better obstacle detection and avoidance by fusing eight distance sensors and the range finder camera. The fusion model is based on Fuzzy Logic fusion technique using MATLAB software. A fuzzy logic system (FLS) is composed of four main parts which are: fuzzifier, rules, inference engine, and defuzzifier. The block diagram of FLS is shown in Figure 2.

The fuzzification stage is the process of converting a set of inputs to fuzzy sets based on defined fuzzy variables and membership functions. According to a set of rules, the inference is made. Finally, at the defuzzification stage, membership functions are used to map every fuzzy output to a crisp output.

**Figure 3:**
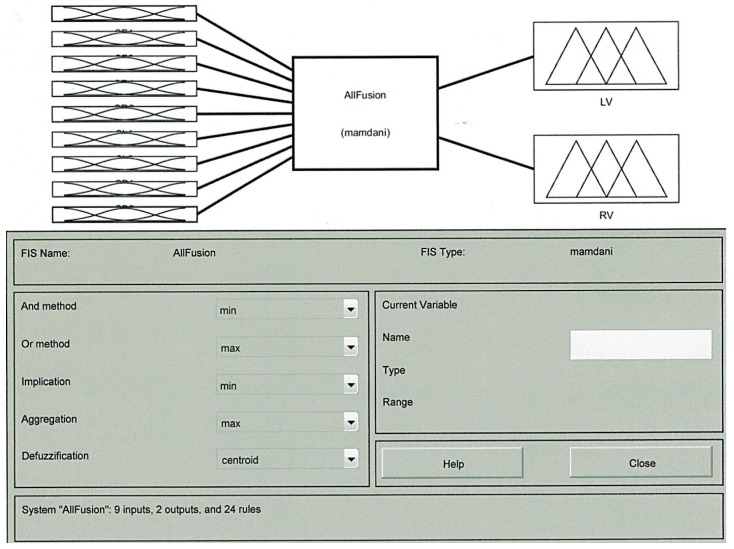
Fuzzy Inference System (FIS) with inputs and outputs.

#### 3.2.1. Fuzzy Sets of the Input and Output

There are nine inputs to the fuzzy logic system and two outputs. The inputs are basically the values of eight distance sensors donated as SF1, SF2, SR1, SR2, SL1, SL2, SB1, and SB2. These sensors measure the amount of light in a range of 0 to 2000 where the threshold is set to 1000 for detected obstacle. The ninth input is the range finder camera value that measures the distance to an obstacle. Two outputs are generated left velocity (LV) and right velocity (RV). Figure 3 shows the Mamdani System using Fuzzy Inference System (FIS) with nine inputs and two outputs.

#### 3.2.2. Membership Functions of the Input and Output

Input variables of distance sensors readings are divided into membership functions which are Obstacle Not Found (OBSNF), and Obstacle Found (OBSF). Both membership functions are a type of trapezoidal-shaped membership function. 

**Figure 4:**
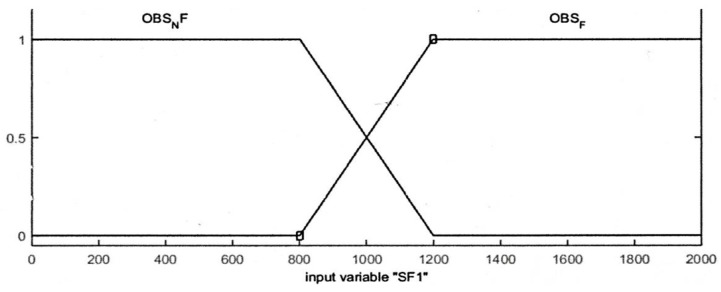
Input membership functions for the distance sensors.

**Figure 5:**
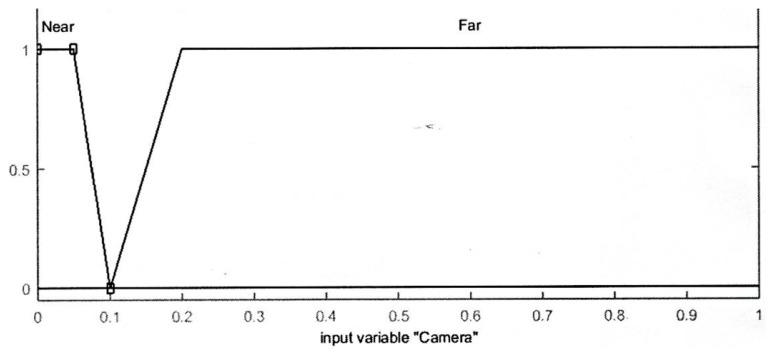
Input membership functions for the camera.

The range for the distance sensor values is [0, 2000] and the threshold is set to 1000 where the value of 1000 or more means an obstacle is found and the robot should avoid it. The input variables of the range finder camera are divided into two trapezoidal-shaped membership functions “Near” and “Far”. The range finder camera measures the distance from the camera to an obstacle in meters. The overall range of the camera input is [0, 1] where 0.1 m is considered as “Near” distance, and collision behavior avoidance should be applied. The input membership functions for distance sensors and the camera are displayed in Figure 4 and Figure 5, respectively. 

Let us assume that x is the sensor value and R is the range of all sensors values where *x∈R*. The trapezoidal-shaped membership function based on four scalar parameters *i, j, k*, and *l*, can be expressed as in Equation (1).

(1) $$\mu_{trap}\left(x:i,j,k,l\right)=max\left(min\left(\frac{x-i}{j-i},1,\frac{l-x}{l-k},0\right)\right)$$

The output variables of left and right velocities of the mobile robot (LV and RV) are divided into two membership functions negative velocity “NEG_V” and positive velocity “POS_V”. The effect and the action of these two memberships on the differential wheels of the robot are summarized as follows:

- If both LV and RV speeds are set to POS_V, then the robot will move forward;
- If LV is set to POS_V and RV is set to NEG_V, then the robot will turn right;
- If LV is set to NEG_V and RV is set to POS_V, then the robot will turn left.

The “NEG_V” is a Z-shaped membership function. This function is represented in Equation (2) where *u* and *q* are two parameters of the most left and most right of the slope.

(2) $$\mu_{z}(x)=\{\begin{array}{cc} 1, & x\leq u\\ 1-2{\left(\frac{x-u}{q-u}\right)}^{2}, & u\leq x\leq \frac{u+q}{2}\\ 2{\left(\frac{x-q}{q-u}\right)}^{2}, & \frac{u+q}{2}\leq x\leq q\\ 0, & x\geq q \end{array}$$

**Figure 6:**
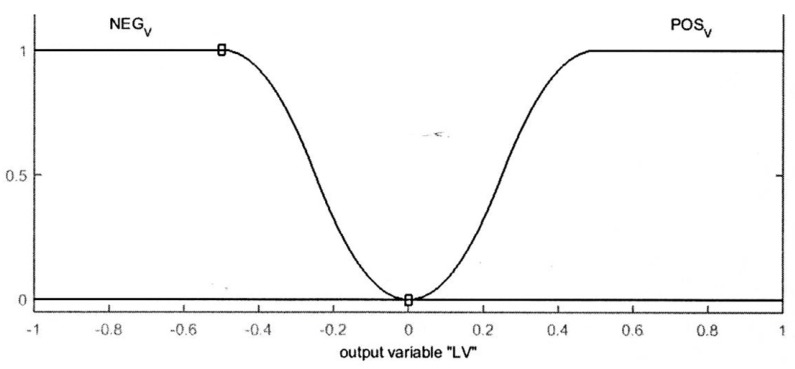
Output membership functions.

In addition, the “POS_V” is an S-shaped membership function where y1and y2 are two parameters of the leftmost and rightmost of the slope. The S-shaped membership function can be expressed as in Equation (3). Figure 6 shows the output membership functions.

(3) $$\mu_{s}(x)=\{\begin{array}{cc} 1, & x\leq y1\\ 2{\left(\frac{x-y1}{y2-y1}\right)}^{2}, & y1\leq x\leq \frac{y1+y2}{2}\\ 1-2{\left(\frac{x-y2}{y2-y1}\right)}^{2}, & \frac{y1+y2}{2}\leq x\leq y2\\ 0, & x\geq y2 \end{array}$$

#### 3.2.3. Designing Fuzzy Rules

Based on the membership functions of the fuzzy set and inputs and outputs, rules are defined. There are 24 rules for collision avoidance of the mobile robot.. We can use AND or OR operations for connecting membership values where the fuzzy AND is the minimum of two or more membership values and OR is the maximum of two or more membership values. Let *μγ* and *μδ* be two membership values, then the fuzzy AND and fuzzy OR are described as in Equations (4) and (5), respectively. In addition, Table 1 lists all the rules with the fuzzy AND operator that express the movement behavior of the mobile robot.

(4) $$\mu_{\gamma }\text{ AND }\mu_{\delta }=\text{ min}\left(\mu_{\gamma },\mu_{\delta }\right)$$

(5) $$\mu_{\gamma }\text{ OR }\mu_{\delta }=\text{ max }\left(\mu_{\gamma },\mu_{\delta }\right)$$

#### 3.2.4. Defuzzification

The last step of designing the fuzzy logic fusion system is the defuzzification process where outputs are generated based on fuzzy rules, membership values, and a set of inputs. The method used for defuzzification is the Centroid method.

**Figure 7:**
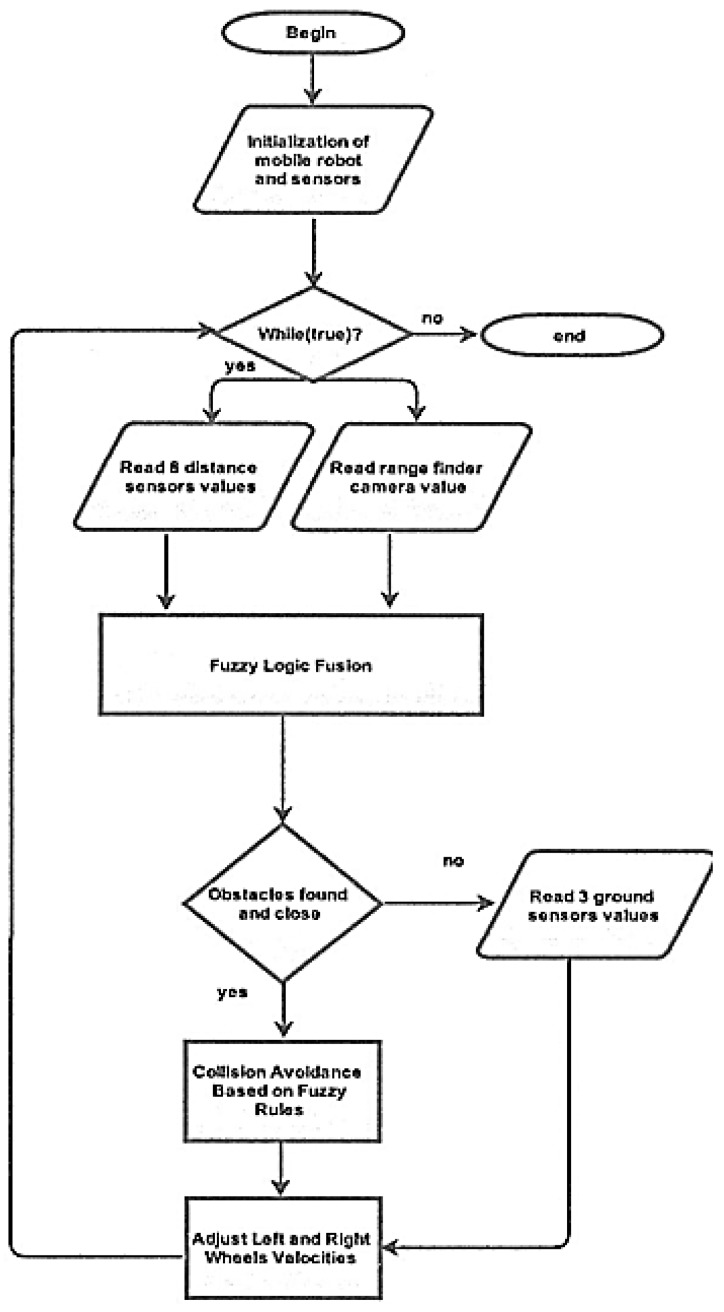
Flowchart of the proposed methodology.

Moreover, the fuzzy logic fusion model was designed for preventing the mobile robot from colliding with any obstacles while following the line. The fusion model composed of nine inputs, two outputs, and 24 rules. Figure 7 demonstrates the proposed methodology. As shown in Figure 7, the initialization of the robot and its sensors is the first step. After that, the distance sensors and camera values are fed into the fuzzy logic fusion system for obstacle detection and distance measurements. If an obstacle is found, the mobile robot will adjust its speed for turning left or right based on the position of the obstacle. The decision is made based on defined fuzzy rules. After avoiding the obstacle, the mobile robot should continue following the line by obtaining ground sensor values and finally adjust its speed accordingly. On the other hand, if there is no obstacle detected, the mobile robot should follow the line while it checks for obstacles to avoid at each time step.

**Table 1:** Fuzzy logic rules.

No	SF1	SF2	SR1	SR2	SL1	SL2	SB1	SB2	LV	RV
1	OBS_F	OBS_F	OBS_NF	OBS_NF	OBS_NF	OBS_NF	OBS_NF	OBS_NF	NEG_V	POS_V
2	OBS_F	OBS_NF	OBS_NF	OBS_NF	OBS_NF	OBS_NF	OBS_NF	OBS_NF	NEG_V	POS_V
3	OBS_NF	OBS_F	OBS_NF	OBS_NF	OBS_NF	OBS_NF	OBS_NF	OBS_NF	NEG_V	POS_V
4	OBS_NF	OBS_NF	OBS_NF	OBS_NF	OBS_F	OBS_F	OBS_NF	OBS_NF	POS_V	NEG_V
5	OBS_NF	OBS_NF	OBS_NF	OBS_NF	OBS_F	OBS_NF	OBS_NF	OBS_NF	POS_V	NEG_V
6	OBS_NF	OBS_NF	OBS_NF	OBS_NF	OBS_NF	OBS_F	OBS_NF	OBS_NF	POS_V	NEG_V
7	OBS_NF	OBS_NF	OBS_F	OBS_F	OBS_NF	OBS_NF	OBS_NF	OBS_NF	NEG_V	POS_V
8	OBS_NF	OBS_NF	OBS_F	OBS_NF	OBS_NF	OBS_NF	OBS_NF	OBS_NF	NEG_V	POS_V
9	OBS_NF	OBS_NF	OBS_NF	OBS_F	OBS_NF	OBS_NF	OBS_NF	OBS_NF	NEG_V	POS_V
10	OBS_NF	OBS_NF	OBS_NF	OBS_NF	OBS_NF	OBS_NF	OBS_F	OBS_F	POS_V	POS_V
11	OBS_NF	OBS_NF	OBS_NF	OBS_NF	OBS_NF	OBS_NF	OBS_F	OBS_NF	POS_V	POS_V
12	OBS_NF	OBS_NF	OBS_NF	OBS_NF	OBS_NF	OBS_NF	OBS_NF	OBS_F	POS_V	POS_V
13	OBS_F	OBS_NF	OBS_NF	OBS_NF	OBS_F	OBS_F	OBS_NF	OBS_NF	POS_V	NEG_V
14	OBS_F	OBS_NF	OBS_NF	OBS_NF	OBS_F	OBS_NF	OBS_NF	OBS_NF	POS_V	NEG_V
15	OBS_F	OBS_NF	OBS_NF	OBS_NF	OBS_NF	OBS_F	OBS_NF	OBS_NF	POS_V	NEG_V
16	OBS_F	OBS_NF	OBS_F	OBS_F	OBS_NF	OBS_NF	OBS_NF	OBS_NF	NEG_V	POS_V
17	OBS_F	OBS_NF	OBS_F	OBS_NF	OBS_NF	OBS_NF	OBS_NF	OBS_NF	NEG_V	POS_V
18	OBS_F	OBS_NF	OBS_NF	OBS_F	OBS_NF	OBS_NF	OBS_NF	OBS_NF	NEG_V	POS_V
19	OBS_NF	OBS_F	OBS_NF	OBS_NF	OBS_F	OBS_F	OBS_NF	OBS_NF	POS_V	NEG_V
20	OBS_NF	OBS_F	OBS_NF	OBS_NF	OBS_F	OBS_NF	OBS_NF	OBS_NF	POS_V	NEG_V
21	OBS_NF	OBS_F	OBS_NF	OBS_NF	OBS_NF	OBS_F	OBS_NF	OBS_NF	POS_V	NEG_V
22	OBS_NF	OBS_F	OBS_F	OBS_F	OBS_NF	OBS_NF	OBS_NF	OBS_NF	NEG_V	POS_V
23	OBS_NF	OBS_F	OBS_F	OBS_NF	OBS_NF	OBS_NF	OBS_NF	OBS_NF	NEG_V	POS_V
24	OBS_NF	OBS_F	OBS_NF	OBS_F	OBS_NF	OBS_NF	OBS_NF	OBS_NF	NEG_V	POS_V

## 4. Simulation and Real Time Implementation for Mobile Robot Navigation

**Figure 8:**
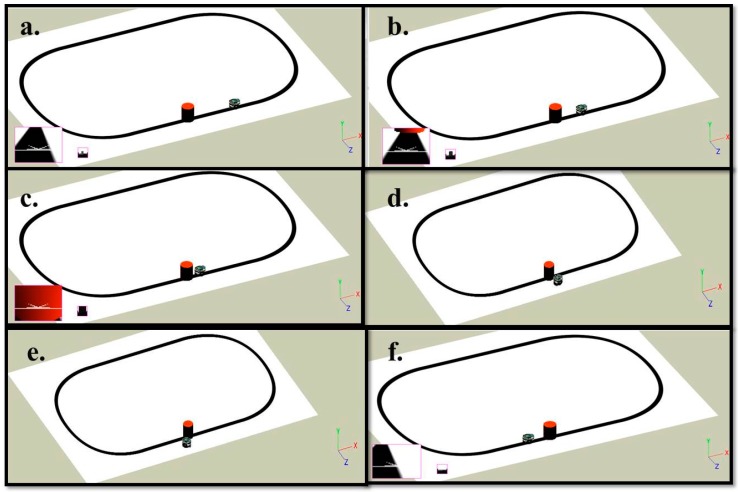
Simulation snapshots of one robot and one obstacle. (**a**) The beginning of the simulation; (**b**) The robot moves forward; (**c**) The robot detects an obstacle on its way; (**d**) The robot avoids the obstacle and turns left; (**e**) The robot tries to find the path again; (**f**) The robot continues following the line.

**Figure 9:**
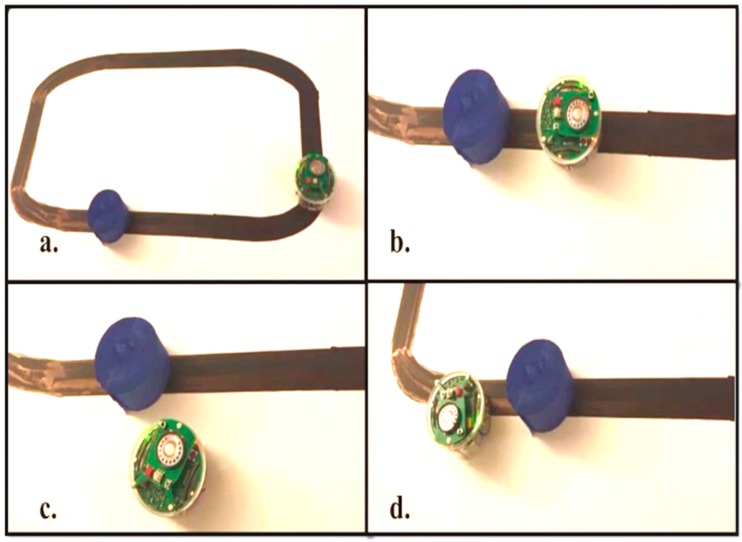
Snapshots of the real time experiment of one robot and one obstacle. (**a**) The beginning of the real time experiment; (**b**) The robot detects an obstacle on its way; (**c**) The robot avoids the obstacle and turns left; (**d**) The robot finds its path again and continues following it.

The environment and the robot are modeled using the Webots Pro simulator for mobile robot collision free navigation. The e-puck used has eight distance sensors which are infrared sensors, camera, three ground sensors, and GPS. The e-puck first senses the environment for possible collisions by using the distance sensors and the range finder camera readings. If there is no obstacle detected, the e-puck follows a black line drawn on a white surface. Snapshots of the simulation and real time experiment for one robot detecting and avoiding an obstacle while following the line are depicted in Figure 8 and Figure 9, respectively. Both figures show the environment with one mobile robot moving forward until it detects an obstacle. After the detection of the obstacle, all readings are fed into the proposed fuzzy logic fusion model and, based on the defined fuzzy rules, the e-puck will turn accordingly by adjusting the left and right wheels velocities. After that, the e-puck will continue moving forward and follow the line.

**Figure 10:**
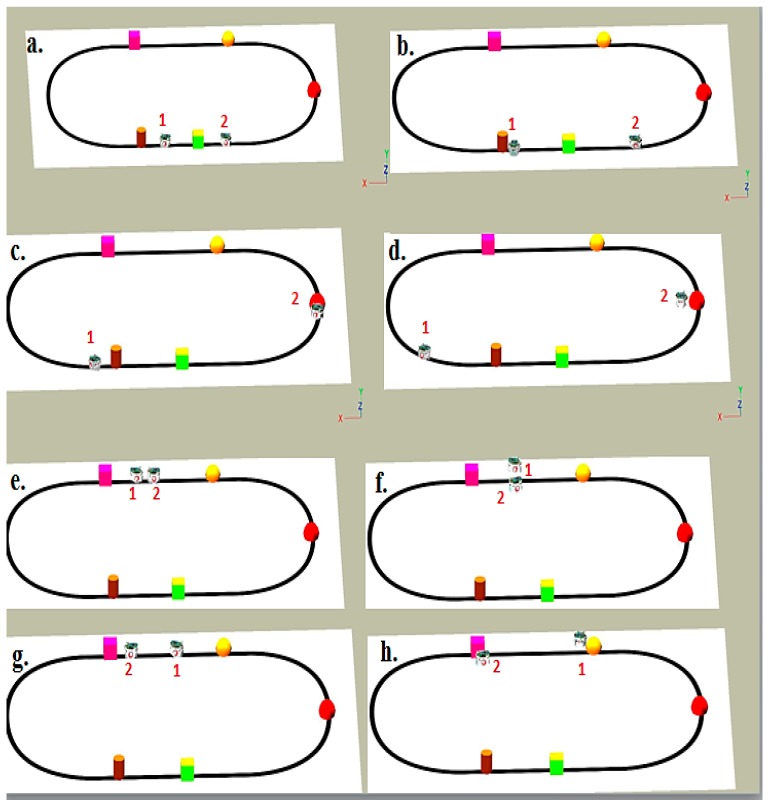
Simulation snapshots for multiple robots and obstacles. (**a**) Both robots at the beginning of the simulation; (**b**) Robot 1 detetcts an obstacle and Robot 2 moves forward; (**c**) Robot 1returns to the line and Robot 2 detects an obstacle; (**d**) Robot 1 continues following the line, and Robot 2 avoids the obstacle and moves around it; (**e**) Robot 1 and 2 detetc each other as dynamic obstacles; (**f**) Both robots avoid each other; (**g**) Both robots have successfully avoided each other and returned to the line; (**h**) Both robots detect other obstacles again.

**Figure 11:**
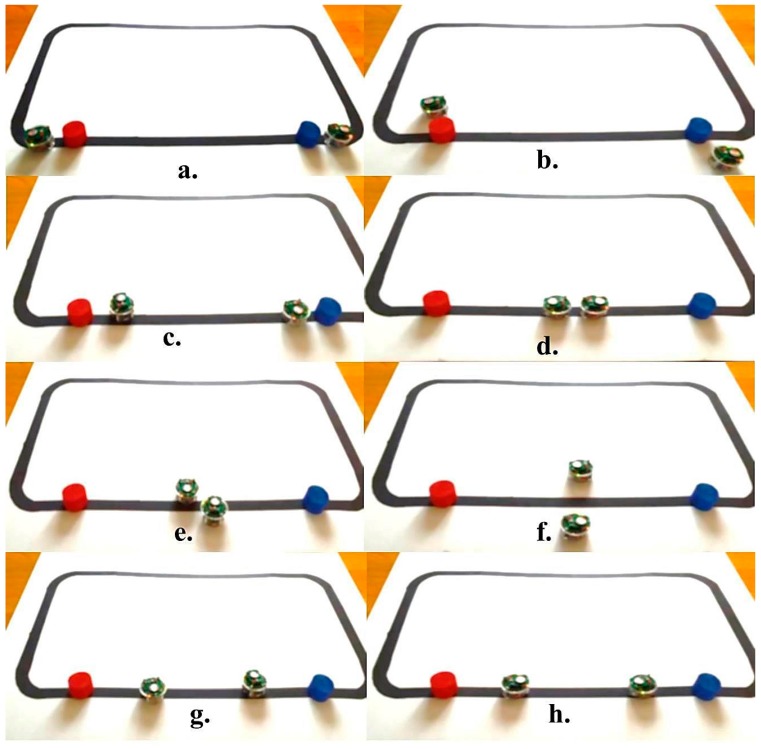
Real time experiment for multiple robots and obstacles. (**a**) Both robots detect obstacles; (**b**) Both robots avoid the obstacles; (**c**) Both robots return to the line; (**d**) Both robots detect each other as dynamic obstacles; (**e**) Both robots avoid each other; (**f**) Both robots have successfully avoided each other; (**g**) Both robots return to the line; (**h**) Both robots continue following the line.

In addition, a more complex environment with various obstacles in different shapes and sizes has been modeled and tested through simulation and real time experiments. Figure 10 and Figure 11 present snapshots of the simulation and real time experiments for two robots following a black line and avoiding different types of obstacles. As shown in these figures, both robots face and detect each other successfully. Each robot tries to avoid the other by adjusting its speeds and returns to follow the line. These robots are considered as dynamic obstacles to each other. 

## 5. Results and Investigation of the Proposed Model

### 5.1. Data Collection and Analysis

This section presents the sensors values obtained from the simulation at different time steps. Three different scenarios have been presented. The first one is a simple environment containing one obstacle and one robot, whereas the second one is a more complex environment that has more static and dynamic obstacles and two robots. The third scenario has more cluttered obstacles, which makes it a more challenging environment for the mobile robot navigation.

#### 5.1.1. First Scenario

Table 2 and Table 3 show the sensors values before and after applying the fuzzy logic fusion model in a simple environment at three different simulation times. At T1, the robot is far away from the obstacle; at T2, the robot is very close to the obstacle, and, at T3, the robot has passed the obstacle successfully. When distance sensor values are below the threshold (1000), it means that there is no obstacle detected. However, when it goes above the threshold, it means that there is an obstacle and the robot needs to adjust its movement. As shown in Table 2, the front distance sensor SF1 has a value of 1159.18, which is higher than the threshold value at T2 and occurs before applying the fuzzy logic fusion method.

In addition, both front distance sensors SF1 and SF2 have higher values than the threshold, which are 1127.19 and1077.76, respectively, at T2 where the fuzzy logic technique is applied. Again, this means that there is an obstacle detected.

**Table 2:** Distance sensor values before and after implementing the fusion model.

Distance Sensor	Without Fuzzy Logic Fusion			With Fuzzy Logic Fusion		
	T1 = 5	T2 = 15	T3 = 41	T1 = 5	T2 = 22	T3 = 41
SF1	14.71	1159.18	64.05	11.73	1127.19	72.72
SF2	35.19	220.33	53.72	48.48	1077.76	54.80
SR1	59.53	24.41	40.20	45.69	35.14	42.40
SR2	31.55	26.77	4.68	31.54	28.00	28.47
SL1	23.78	36.03	59.58	21.23	58.54	31.90
SL2	59.49	18.59	33.88	13.40	38.10	72.51
SB1	19.99	91.65	14.64	22.21	56.23	59.42
SB2	46.62	13.29	5.08	66.13	30.13	47.91

**Table 3:** Distance measurements by camera before and after implementing the fusion model.

Range Finder Camera	Without Fuzzy Logic Fusion			With Fuzzy Logic Fusion		
	T1 = 5	T2 = 13	T3 = 41	T1 = 5	T2 = 22	T3 = 41
Distance to Obstacle in Meter	0.337	0.097	x	0.339	0.040	x

**Table 4:** Ground sensor values at different simulation times.

Simulation Time	Ground Sensor 1	Ground Sensor 2	Ground Sensor 3	Delta
5	287.83	256.58	330.61	42.77
10	271.00	250.32	327.13	56.12
13	323.11	307.06	353.36	30.25
41	266.31	290.37	277.68	11.36

Table 3 shows the distance to obstacles measured in meters by the range finder camera at various simulation times T1, T2, and T3. As represented in Table 3, once the robot approaches the obstacle, the distance between the robot and the obstacle is decreased. At T2, the distance between the robot and the obstacle, where the obstacle is firstly detected by the camera and before applying the fuzzy logic method is 0.097 m where it is only 0.040 m after applying the fusion model. The camera can measure the distance up to one meter ahead. At T3, the camera could not measure the distance to an obstacle because it did not find any obstacles within its range. 

In addition, Table 4 shows the three ground sensors values used for the line following approach at different simulation times. It also shows the delta values which are the difference between the left and right ground sensors. Delta values are used to adjust the robot’s left and right speeds to follow the line.

Finally, Table 5 shows the robot’s position, orientation, and velocities. The position of the robot has been obtained through the GPS sensor. The position and orientation of the robot according to Webots global coordinates system. As shown in Table 5, when the robot detects an obstacle at a time 22 s of the simulation, its left and right velocities are adjusted. The negative left velocity and the positive right velocity means that the robot is turning at the left direction to avoid the obstacle. At a time of 38 s, the robot has avoided the obstacle and turned right to continue following the line.

**Table 5:** The position, orientation, and velocities of the robot in a simple environment.

Simulation Time in Seconds	Position			Rotation Angle in Degree *θ*	Left Wheel Velocity	Right Wheel Velocity
	x	y	z			
**5**	0.34	0.05	1.24	−92.24	270.24	229.76
**22**	0.08	0.05	1.26	−165.58	−200.65	400.23
**30**	0.03	0.05	1.33	−100.87	200.65	189.44
**38**	−0.06	0.05	1.34	−36.04	305.34	−199.72
**41**	−0.14	0.05	1.24	−101.62	213.16	286.84

#### 5.1.2. Second Scenario

**Figure 12:**
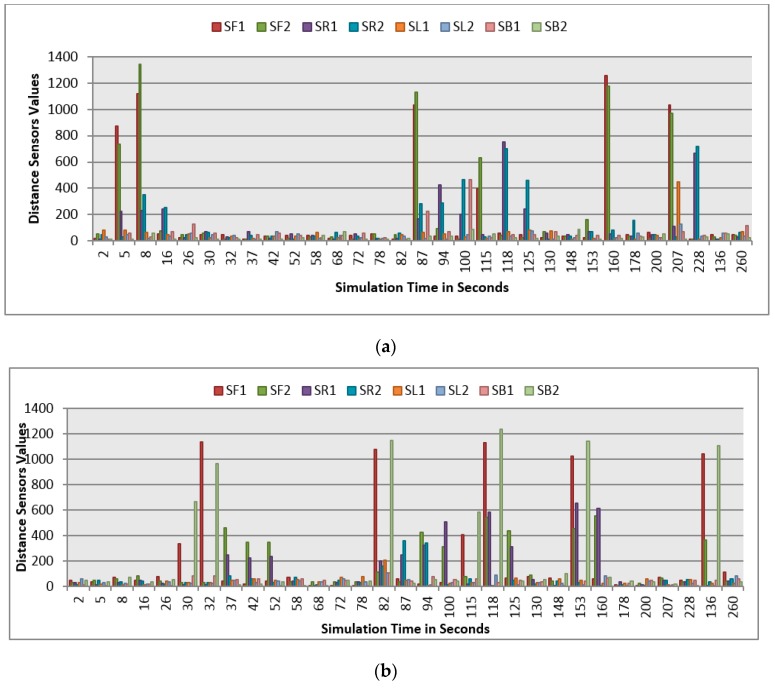
Distance sensor values for both robots at different simulation times. (**a**) Distance sensors readings at different simulation times for Robot 1; (**b**) Distance sensor readings at different simulation times for Robot 2.

**Figure 13:**
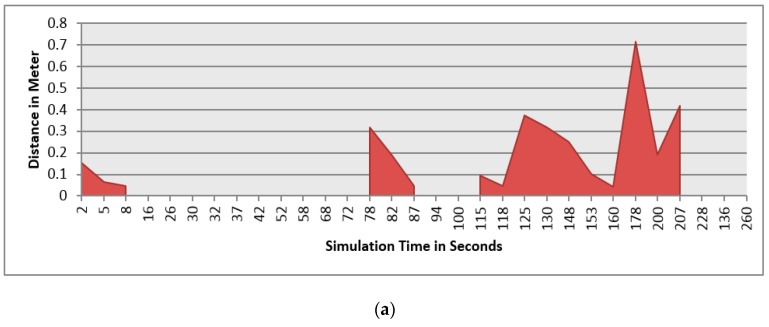

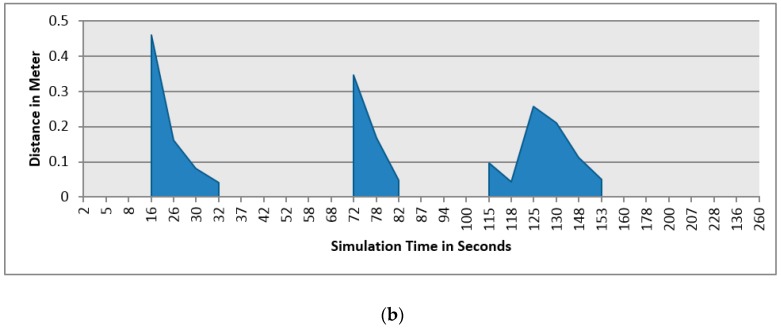
Distance to obstacles for both robots. (**a**) Distance to obstacles in meters for Robot 1; (**b**) Distance to obstacles in meters for Robot 2.

**Figure 14:**
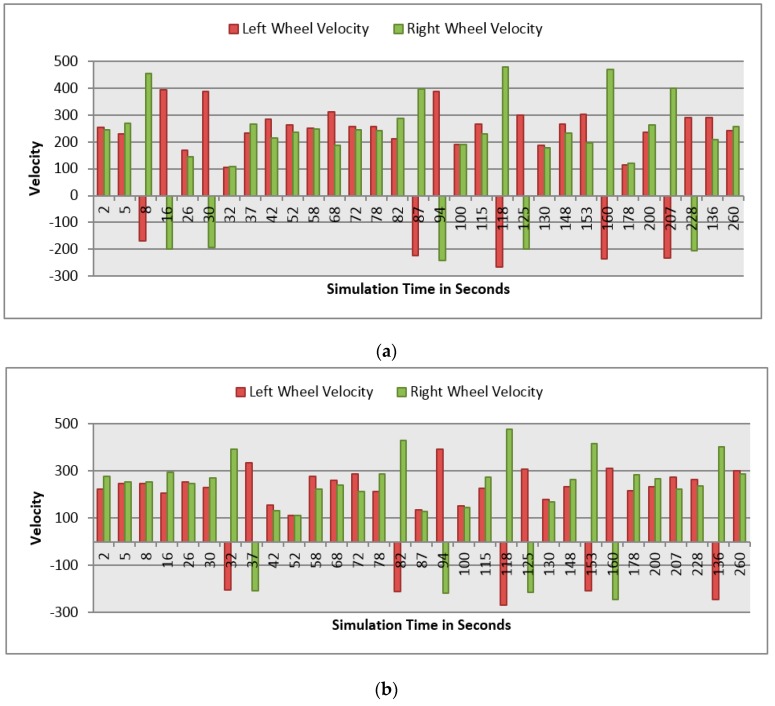
Left and right velocities for both robots at different simulation times. (**a**) Left and right velocities for Robot 1; (**b**) Left and right velocities for Robot 2.

This section demonstrates the proposed model in a more complex environment where it is composed of a number of obstacles in different sizes and shapes. Two robots are running in this scenario where both are avoiding obstacles and each other as well. Each robot considers the other as a dynamic obstacle. As presented in Figure 10, two robots (1 and 2) in opposite directions are following the line and overtaking obstacles. Figure 12, Figure 13 and Figure 14 show all distance sensor readings, distance to obstacles in meters, and left and right velocities through the entire loop for the two robots, respectively. In Figure 13, the distances to obstacles are obtained by the range finder camera where sometimes the obstacle is either in a distance greater than one meter or it is outside the camera field of the view. In later cases, the camera cannot measure the distances between the robots and the obstacles, which explains the gabs in Figure 13a,b. 

**Figure 15:**
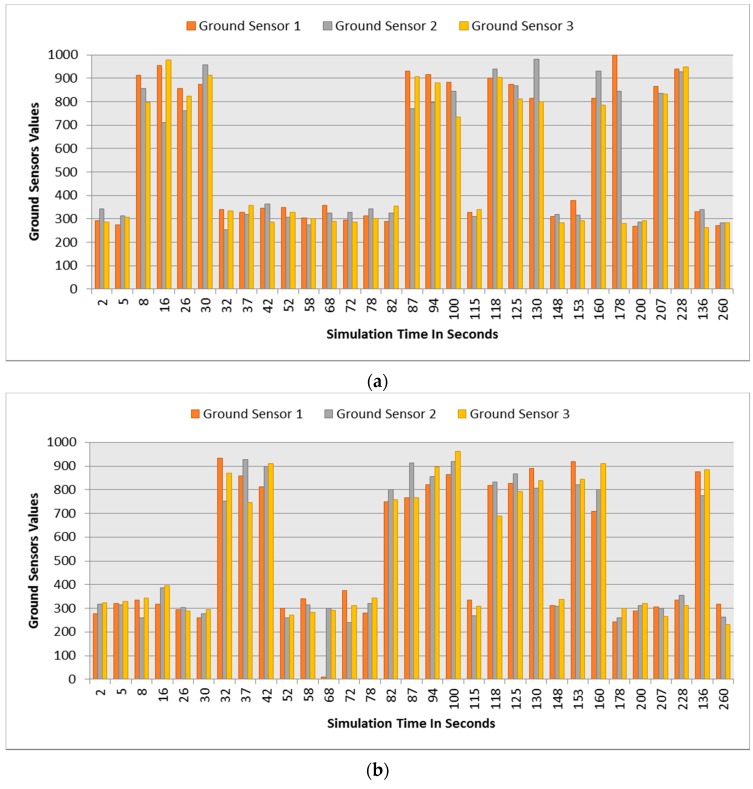
Ground sensors values for both robots at different simulation times. (**a**) Ground sensors values at different simulation times for robot 1; (**b**) Ground sensors values at different simulation times for robot 2.

**Figure 16:**
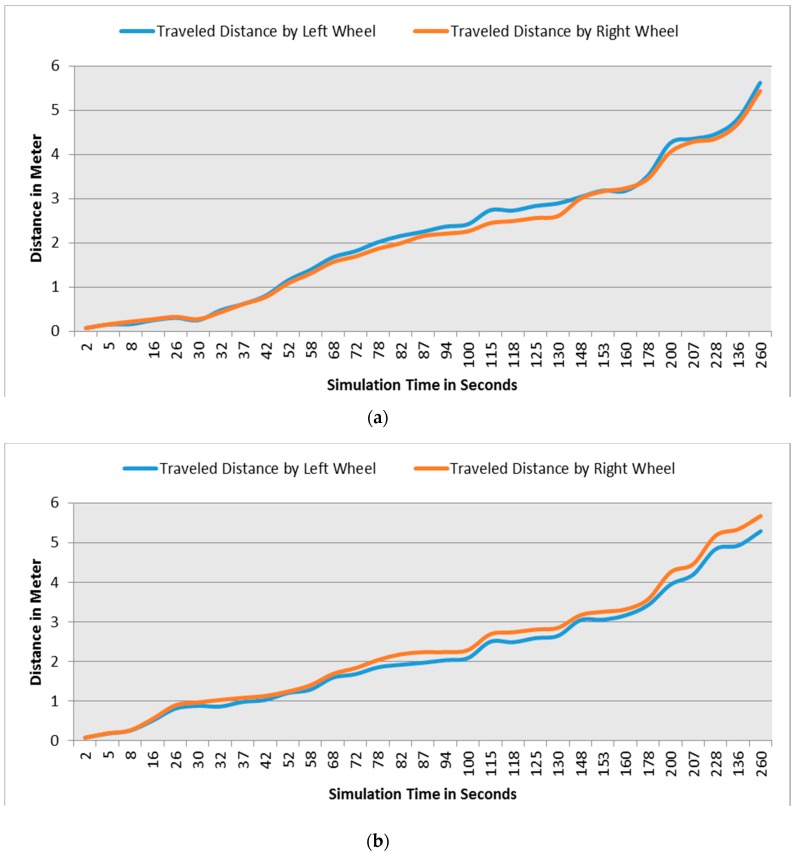
Traveled distance measured by left and right wheels for both robots. (**a**) Distance traveled by left and right wheels for robot 1; (**b**) Distance traveled by left and right wheels for robot 2.

At the beginning of the simulation, the distance between robot 1 and the obstacle is 0.15 m as shown in Figure 13a. At a time of eight seconds, robot 1 detects an obstacle where both front distance sensors (SF1 and SF2) have values greater than the threshold value as presented in Figure 12a. The distance between the robot and the obstacle at a time of eight seconds has been decreased to 0.047 m as shown in Figure 13a. As a result, the robot will adjust its speed accordingly to avoid colliding with the obstacle. Figure 14a, shows the left and right velocities for robot 1. At a time of eight seconds, the left wheel velocity is a negative value (−168) and the right wheel velocity is a positive value (454), which indicates that robot 1 is turning left to avoid collision. At time 16 s, robot 1 is turning right around the obstacle where the left wheel velocity is 395 and the right wheel velocity is −199. After that, the robot will continue following the line until another obstacle is detected. The ground sensor values and traveled distance by left and right wheels for both robots during the entire loop are demonstrated in Figure 15 and Figure 16, respectively. 

Furthermore, at time 115 s, robots 1 and 2 face each other after avoiding a couple of obstacles successfully. At that time, the distance between both robots is approximately 0.096 m. At a time of 118 s, the distance between them reaches 0.044 m*.* Both robots turn in opposite directions to avoid collision. To illustrate, at this time, the speed of robot 2 has been adjusted as shown in Figure 14b. Both robots will get around each other and return to follow the line. The position and orientation of both robots according to Webots global coordinates system are presented in Table 6. 

#### 5.1.3. Third Scenario

**Figure 17:**
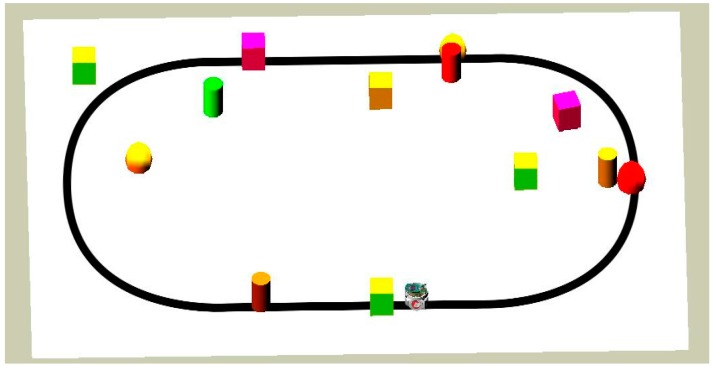
Simulation overview of the mobile robot and the environment.

**Figure 18:**
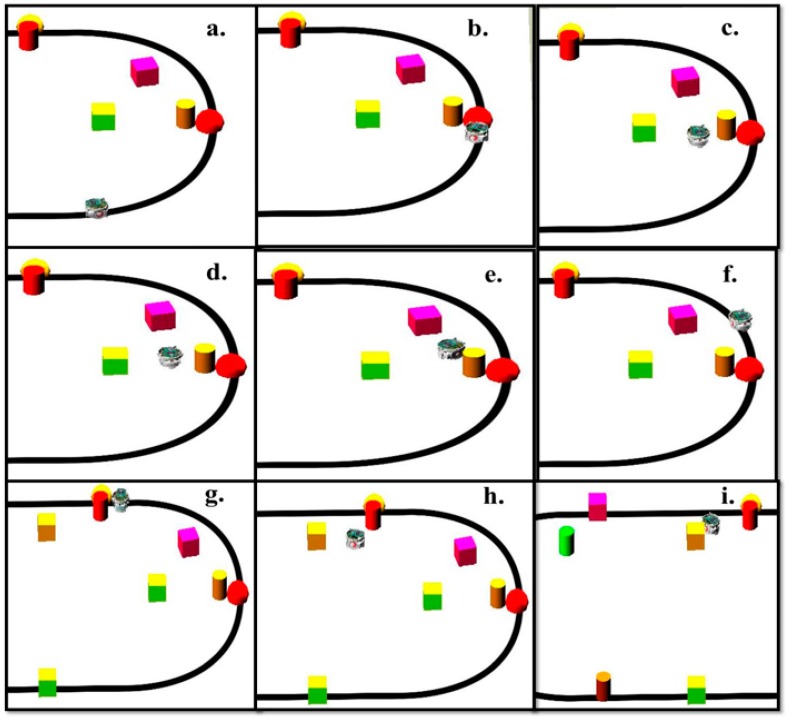
Simulation snapshots at various times. (**a**) The beginning of the simualtion; (**b**) The robot detects an obstacle and turns left; (**c**) The robot moves around the obstacle; (**d**) The robot between multiple scattered obstacles; (**e**) The robot turns right to avoid possible collision; (**f**) The robot returns to the line; (**g**) The robot detects another obstacle on its way; (**h**) The robot avoids obstacles; (**i**) The robot has successfully avoided obstacles.

In this scenario, there are more cluttered obstacles in the environment where it is more challenging for the robot to avoid them. The robot needs to adjust its speed and orientation according to obstacles positions. Figure 17 and Figure 18 represent the simulation of the mobile robot with many cluttered obstacles around. 

**Table 6:** The position and orientation of both robots at different simulation times.

Simulation Time in Seconds	Robot 1				Robot 2			
	Position			Rotation Angle in Degree *θ*	Position			Rotation Angle in Degree *θ*
	x	y	z		x	y	z	
**2**	0.18	0.05	0.15	99.39	−0.20	0.05	0.15	−88.26
**5**	0.27	0.05	0.16	48.17	−0.31	0.05	0.15	−93.40
**8**	0.28	0.05	0.13	16.01	−0.40	0.05	0.15	−93.94
**16**	0.33	0.05	0.07	80.73	−0.62	0.05	0.23	−125.41
**26**	0.43	0.05	0.11	145.54	−0.79	0.05	0.52	−167.63
**30**	0.46	0.05	0.15	145.54	−0.79	0.05	0.59	110.00
**32**	0.47	0.05	0.16	145.54	−0.77	0.05	0.59	110.00
**37**	0.59	0.05	0.17	107.47	−0.72	0.05	0.62	174.72
**42**	0.73	0.05	0.23	127.69	−0.72	0.05	0.68	174.71
**52**	0.90	0.05	0.51	167.23	−0.78	0.05	0.76	−120.48
**58**	0.92	0.05	0.70	179.96	−0.81	0.05	0.83	158.72
**68**	0.87	0.05	0.99	−154.20	−0.71	0.05	1.10	142.67
**72**	0.80	0.05	1.10	−141.02	−0.61	0.05	1.19	121.69
**78**	0.65	0.05	1.21	−111.32	−0.44	0.05	1.25	95.44
**82**	0.54	0.05	1.24	−100.42	−0.34	0.05	1.23	4.69
**87**	0.41	0.05	1.26	−169.79	−0.34	0.05	1.17	4.70
**94**	0.39	0.05	1.34	−105.05	−0.27	0.05	1.13	69.45
**100**	0.31	0.05	1.36	−105.05	−0.21	0.05	1.14	134.26
**115**	0.14	0.05	1.25	261.32	0.05	0.05	1.25	89.07
**118**	0.13	0.05	1.28	−161.27	0.07	0.05	1.22	11.18
**125**	0.10	0.05	1.34	−96.53	0.10	0.05	1.16	75.91
**130**	0.04	0.05	1.34	−96.53	0.15	0.05	1.14	75.91
**148**	−0.01	0.05	1.24	84.02	0.28	0.05	1.24	265.34
**153**	−0.18	0.05	1.24	−101.41	0.36	0.05	1.22	4.43
**160**	−0.19	0.05	1.32	−179.93	0.37	0.05	1.15	69.20
**178**	−0.43	0.05	1.28	−56.51	0.46	0.05	1.25	84.13
**200**	−0.80	0.05	0.86	−9.46	0.88	0.05	0.94	18.56
**207**	−0.83	0.05	0.75	−81.51	0.91	0.05	0.78	6.13
**228**	−0.83	0.05	0.56	53.63	0.48	0.05	0.16	−71.84
**136**	−0.74	0.05	0.37	28.13	0.39	0.05	0.20	−170.91
**260**	−0.17	0.05	0.16	82.96	0.17	0.05	0.15	−94.52

**Figure 19:**
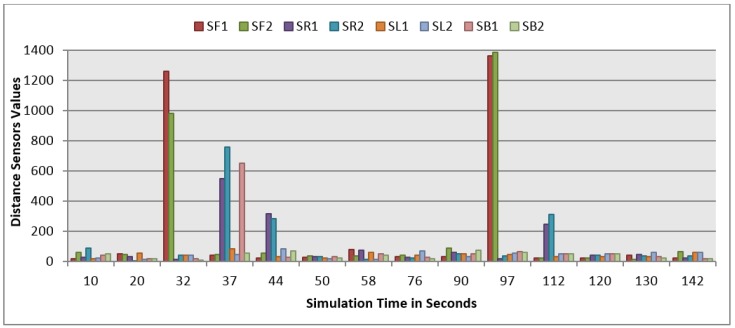
Distance sensor values at different simulation times.

**Figure 20:**
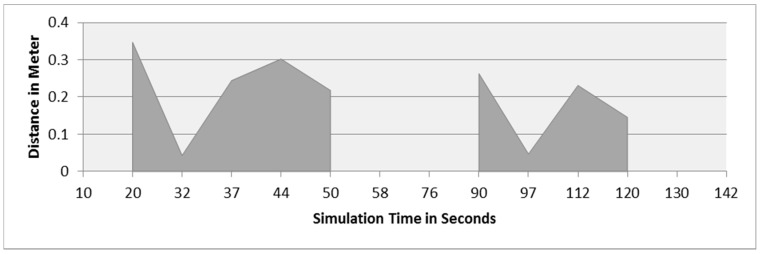
Distance to obstacles in meters at different simulation times.

**Figure 21:**
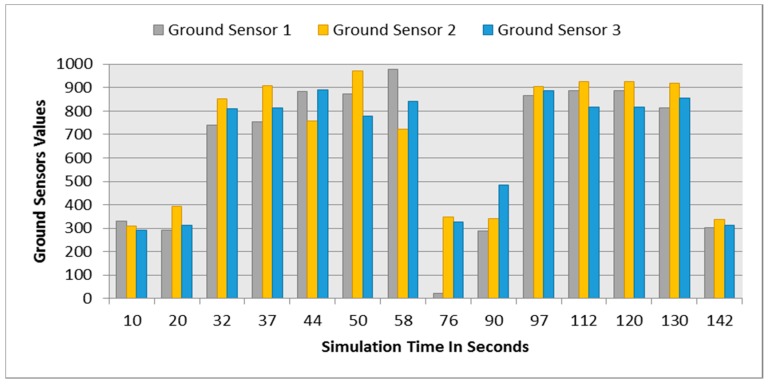
Ground sensor values at different simulation times.

**Figure 22:**
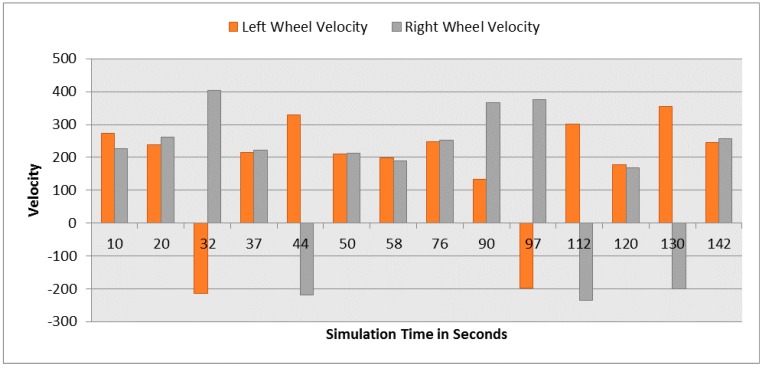
Left and right wheel velocities at different simulation times.

Figure 19 demonstrates the distance sensor values, and Figure 20 shows the distance to obstacles obtained by the range finder camera at various simulation times. At the beginning of the simulation, the robot starts sensing the environment for possible obstacle detection. It also follows the predefined line using the ground sensors. As shown in Figure 18b, the robot turned left due to obstacle presence. At 32 s of the simulation time, SF1 (front distance sensor) reached a value of 1261, which indicates that there is an obstacle detected (Figure 19). In addition, the range finder camera has measured the distance to that obstacle which is 0.043 m as in Figure 20. At 37 s, the robot detects another obstacle on its right side and moves forward as in Figure 18c. Then, the robot gets stuck in between two obstacles and another obstacle in front of it. As shown in Figure 18d, the robot tries to travel in between both obstacles. At 50 s, the distance between the robot and the front obstacle is 0.21 m as in Figure 20. After that, the robot turns right to catch the line again as in Figure 18f. Figure 21 depicts the ground sensor values at different times, and Figure 22 shows the left and right wheels’ velocities of the robot. At a time of 97 s, the robot detects another obstacle on its path and turns left as indicated in Figure 18g and Figure 22. At 112 s, the robot detects an obstacle on its right side, which is very close to the first one and another obstacle at the front as in Figure 18h. Again, the robot tries to move in between both obstacles to recover its path as in Figure 18i. Table 7 summaries the robot’s position and rotation angle in degrees at various simulation times. 

### 5.2. Results and Discussion

**Figure 23:**
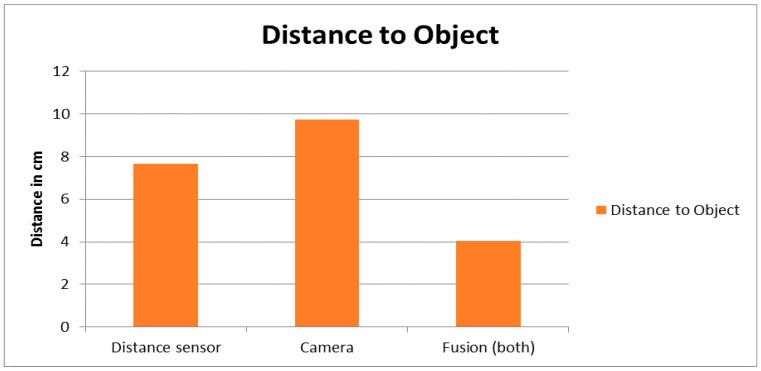
Distance measurements between the robot and the obstacle.

The e-puck has successfully detected different types of obstacles (static and dynamic obstacles) with various shapes and sizes, and avoided them while it was following the line. Different scenarios have been presented with simple, complex, and challenging environments. Distance sensors and camera are used for obstacle detection and distance measurement. The distance sensor can only detect the obstacle when the robot is very close to the obstacle while the camera can detect it up to one meter ahead of the robot. Before applying the proposed fusion model, the distance sensors had detected the obstacle at a distance of 0.076 m from that obstacle to the robot while the camera has detected the obstacle at a distance of 0.097 m between the camera and the obstacle. On the other hand, after implementing the proposed fuzzy logic fusion methodology for collision avoidance behavior, the robot has detected the obstacle at a distance of 0.040 m. Detecting obstacles in a short distance range is very efficient and beneficial, especially in a dynamic environment where the robot quickly detects obstacles just gotten in its way. Figure 23 demonstrates the distance to obstacle measurements by using the distance sensor, or the camera, both with the integration of fusion model. As shown in Figure 23, fusing both sensors outweighs the performance of using each sensor separately.

**Figure 24:**
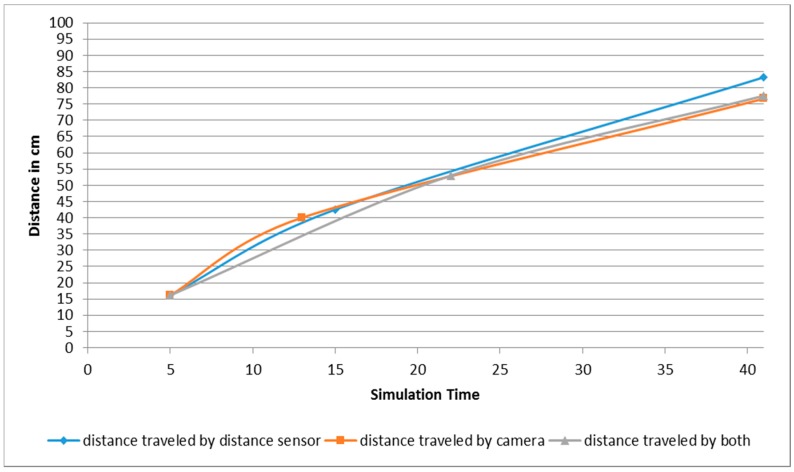
Average of distance traveled by the robot’s differential wheels.

**Figure 25:**
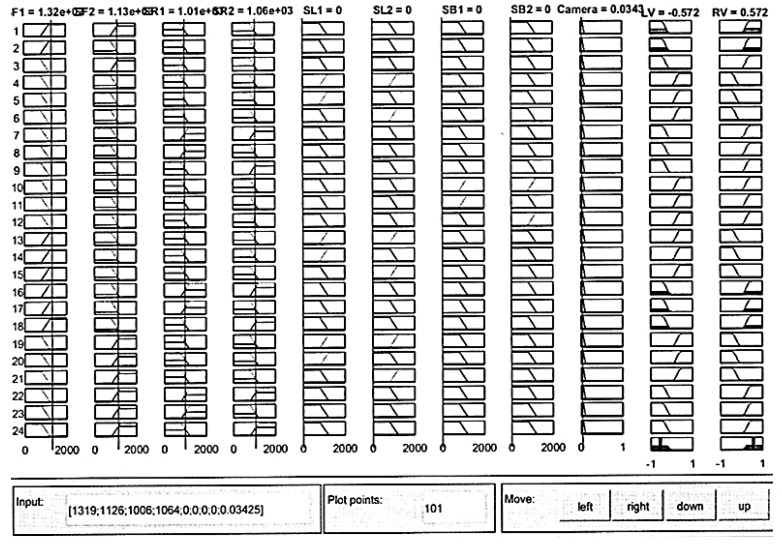
An example of the fusion model at MATLAB’s rules viewer.

Furthermore, the distance traveled by the left and right robot’s differential wheels is observed. As shown in Figure 24, the proposed fusion model has helped in reducing the distance traveled by the robot as opposed to each sensor separately, especially at the beginning of the simulation, which saves more energy, time, and computational load. In addition, an example of the proposed model using the MATLAB rule viewer is presented in Figure 25. In this figure, the sensor values of SF1, SF2, SR1, and SR2 which are the front and right distance sensors are higher than the set threshold. As a result, there are obstacles detected at the front and right sides of the robot’s position. As shown in Figure 25, LV has a negative value and RV has a positive value, which means that the robot turns left due to the presence of obstacles at the front and right sides.

In addition, our approach aims at following the robot along a predefined path (black line on a white surface) while avoiding multiple obstacles on its way such as static, dynamic, and cluttered obstacles. Applying the fuzzy logic fusion has successfully reduced the distance traveled by the robot’s wheels and minimized the distance between the robot and the obstacle detected as compared to a non-fuzzy logic approach, which is beneficially in a dynamic environment. Unlike other optimal planners such as Dijkstra, our approach does not focus on the shortest route and time taken to a specific target, the terrain characteristic, and energy of control actions.

**Table 7:** Summaries of the robot’s position and rotation angle at various simulation times.

Simulation Time in Seconds	Position			Rotation Angle in Degree *θ*
	x	y	z	
10s	−0.46	0.05	0.16	−101.09
20s	−0.72	0.05	0.33	−147.02
32s	−0.79	0.05	0.59	109.58
37s	−0.71	0.05	0.62	109.58
44s	−0.65	0.05	0.65	174.30
50s	−0.64	0.05	0.74	175.88
58s	−0.68	0.05	0.80	−119.31
76s	−0.78	0.05	0.93	171.94
90s	−0.52	0.05	1.23	107.53
97s	−0.34	0.05	1.23	9.67
112s	−0.27	0.05	1.07	74.42
120s	−0.19	0.05	1.05	92.64
130s	−0.11	0.05	1.14	139.23
142s	0.05	0.05	1.24	81.03

## 6. Conclusions

In this article, a multisensory fusion based model was proposed for collision avoidance and path following the mobile robot. Eight distance sensors and a range finder camera were used for the collision avoidance behavior where three ground sensors were used for the line following approach. In addition, a GPS was used to obtain the robot’s position. The fusion model designed is based on the fuzzy logic inference system, which is composed of nine inputs, two outputs, and 24 fuzzy rules. Multiple membership functions for inputs and outputs are developed. The proposed methodology has been successfully tested in the Webots Pro simulator and with the real time experiment. Different scenarios have been presented with simple, complex, and challenging environments. The robot detected static and dynamic obstacles with different shapes and sizes in a short distance range, which is very efficient in dynamic environment. The distance traveled by the robot was reduced using the fusion model, which reduces energy and computational consumptions and time.
